# Surface Structuring Combined with Chemical Surface Functionalization: An Effective Tool to Manipulate Cell Adhesion

**DOI:** 10.3390/molecules24050909

**Published:** 2019-03-05

**Authors:** Sarah M. Elsayed, Stefan Paschke, Sibylle J. Rau, Karen Lienkamp

**Affiliations:** Freiburg Center for Interactive Materials and Bioinspired Technologies (FIT) and Department of Microsystems Engineering (IMTEK), Albert-Ludwigs-Universität, Georges-Köhler-Allee 105, 79110 Freiburg, Germany; sarah.mahmoud@imtek.uni-freiburg.de (S.M.E.); stefan.paschke@imtek.uni-freiburg.de (S.P.); sibylle.rau@uniklinik-freiburg.de (S.J.R.)

**Keywords:** colloidal lithography, polymer surfaces, surface functionalization, surface-cell interactions, surface structuring, tissue engineering

## Abstract

In this study, we investigate how a surface structure underneath a surface-attached polymer coating affects the bioactivity of the resulting material. To that end, structured surfaces were fabricated using colloidal lithography (lateral dimensions: 200 nm to 1 µm, height ~15 to 50 nm). The surface structures were further functionalized either with antimicrobial, cell-adhesive polycations or with protein-repellent polyzwitterions. The materials thus obtained were compared to non-functionalized structured surfaces and unstructured polymer monolayers. Their physical properties were studied by contact-angle measurements and atomic force microscopy (AFM). Protein adhesion was studied by surface plasmon resonance spectroscopy, and the antimicrobial activity against *Escherichia coli* bacteria was tested. The growth of human mucosal gingiva keratinocytes on the materials was analyzed using the Alamar blue assay, optical microscopy, and live-dead staining. The data shows that the underlying surface structure itself reduced protein adhesion and also bacterial adhesion, as evidenced by increased antimicrobial activity. It also enhanced cell adhesion to the surfaces. Particularly in combination with the adhesive polycations, the surfaces increased the cell growth compared to the unstructured reference materials. Thus, functionalizing structured surfaces with adhesive polymer could be a valuable tool for improved tissue integration.

## 1. Introduction

Chemical and physical surface properties, such as surface chemistry, interfacial energy, roughness or structuring determine to a large extent how organisms interact with materials [1,2,3,4,5]. Thus, by deliberately designing surface properties, the interaction of matter with life can be directed. This has implications for all technical products that come into contact with mammalian cells, bacteria, yeasts or even plant cells like algae—for example medical devices, ship hulls, water transportation and purification systems, and building parts. Through customized surface properties, desired interactions can be enhanced (e.g., when tailor-made implant surfaces lead to successful tissue integration), and undesired interactions can be suppressed (e.g., when protein-repellent coatings suppress algae attachment on a ship hull). Surface functionalization thus is a key tool for successful product development.

In the context of biomedical devices, one of the most pressing problems is undesired biofilm formation. When bacteria adhere to a medical device surface, they proliferate and can form a mature biofilm within 24 h [6]. Several approaches to prevent biofilm formation are based on surface functionalization with polymer coatings [5,7,8,9]. This strategy has the advantage that only the interfacial properties of the device are changed, while the bulk properties of the material from which the device has been fabricated remain unchanged. For example, coatings made from “fouling-release” materials such as PDMS enable removal of adhered bacteria by low shear forces, while “non-fouling” coatings made from hydrophilic polymers such as poly(ethylene glycol) or polyzwitterions prevent protein adhesion [5,9]. Coatings made from contact-killing polycations, on the other hand, kill adhered bacteria, yet on the expense of the bacterial debris contaminating the surface, which is then becoming vulnerable to biofilm formation [7,8]. In the context of implants, a “race on the surface” of bacterial biofilm formation versus adhesion of mammalian cells takes place as soon as a biomaterial is put into the body [10,11,12]. In that context, chemical surface modification and microstructuring can reduce bacterial adhesion and proliferation, and assist successful tissue integration [12].

**Figure 1:**
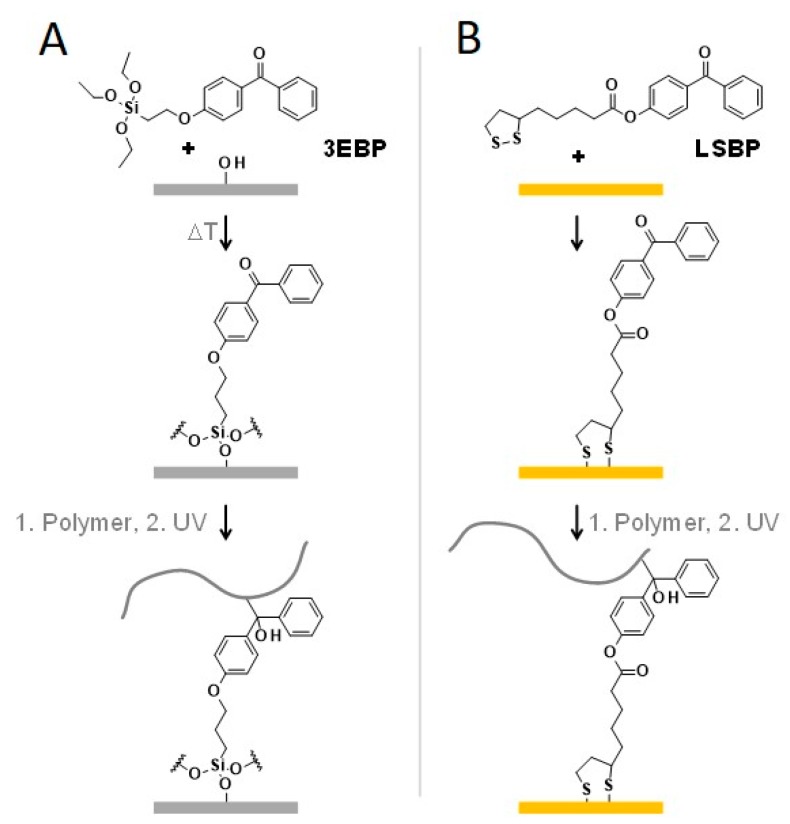
Anchor groups for covalent attachment of polymers on surfaces. (**A**) 4-(3-triethoxysilane propoxyl) benzophenone (**3EBP**) for substrates with OH groups, (**B**) 1,2-dithiolane-3-pentanoic acid 4-benzophenone ester (**LS-BP**) for gold substrates.

Covalent attachment of a polymer coating to the surface enhances the coating stability and prevents delamination. Thus, chemical surface functionalization of materials with suitable anchor groups is an important prerequisite to the actual coating formation. In that context, surface modification with UV-reactive benzophenone-functionalized silanes or disulfides [13,14,15] is a useful tool to immobilize a large variety of polymers on different substrates (Figure 1). When benzophenone is UV irradiated at 254 nm or 365 nm, it unspecifically reacts with nearby C-H bonds via C,H insertion reactions (Figure 1) [3]. Although limitations of the method have also been reported [16], these benzophenone compounds are versatile and easy-to use anchor groups for many applications.

In particular, the silanization agent 4-(3-triethoxysilane propoxyl)benzophenone (**3EBP**, Figure 1a) [14] can be used to attach polymers to surfaces that carry OH groups or that can be oxidized (e.g., by flaming or plasma cleaning). This includes the important laboratory model surface silicon, different kinds of glass, and many metals, ceramics and polymers. The technique has been meanwhile demonstrated even on technical products like polyurethane wound foams [17]. When heating **3EBP**, it forms a thin network-like layer by condensation reactions involving the surface OH groups and neighboring **3EBP** molecules. For gold substrates, the anchor group 1,2-dithiolane-3-pentanoic acid 4-benzophenone ester (**LS-BP**, Figure 1b) can be used. Gold is typically used in the form of a thin layer that has been evaporated onto another substrate, and is important in the context of surface plasmon resonance spectroscopy [18] and microfabrication [19]. Besides chemical surface modification, micro- and nanostructuring methods have been established as additional tools to control and direct cellular interaction with surfaces [1,4,20,21,22,23,24,25,26]. In particular, with these structures, it is possible to control and modify bacterial adhesion and proliferation [27,28,29].

In this work, we combined surface structuring and chemical surface functionalization steps to obtain a set of materials with defined surface chemistry and topography. We then exposed these materials to proteins, bacteria, and human cells, and thereby probed how combined chemical and topographical surface features affect these “life-matter-interactions”. In a previous publication [30], we used the same fabrication technique to obtain bifunctional materials consisting of contact-killing antimicrobial polymer patches made from ‘synthetic mimics of antimicrobial peptides’ (SMAMPs) [31,32] and protein-repellent polymer patches made from polyzwitterionic polysulfobetaines (PSB). We investigated the effect of the polymer patch sizes on the antimicrobial activity and protein-adhesion of these surfaces [30]. While the impact of the polymer patches themselves was quite well understood in that study, one question remained unresolved: What is the effect of the underlying surface pattern itself on bioactivity? Answering this question is the aim of the here presented paper.

## 2. Results

### 2.1. Surface Design and Fabrication

**Figure 2:**
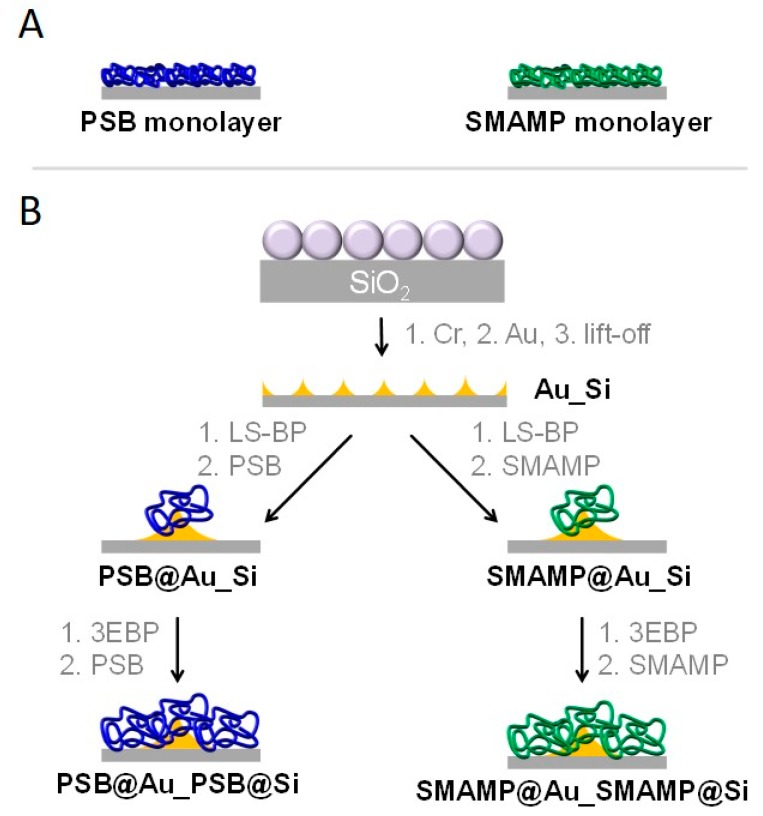
Target materials. (**A**) Homogeneous PSB and SMAMP monolayers. (**B**) Fabrication of polymer monolayers with underlying surface pattern. In the first step, the gold islands were functionalized with **LS-BP**, then with either of the polymers. In the next step, the silicon background was functionalized with **3EBP**, then with the same polymers.

The aim of this paper was to find out how an underlying surface pattern with lateral dimensions between 200 nm and 1 µm and a height between ~15–50 nm affects the bioactivity of polymer-functionalized surfaces. In particular, the bioactivity of unstructured polymer monolayers made from either the antimicrobial, polycationic SMAMP (**SMAMP monolayer**, Figure 2a), or the protein-repellent, polyzwitterionic PSB (**PSB monolayer**, Figure 2a) was compared to structured monolayers made from the same materials (**SMAMP@Au_SMAMP@Si** or **PSB@Au_PSB@Si**, respectively, Figure 2b). The unstructured monolayers were fabricated by spin coating the respective polymers onto **3EBP**-functionalized silicon wafers, followed by UV irradiation [33]. The structured layers were obtained by colloid lithography [34]. First, a monolayer of polystyrene colloids (with either 200 nm, 500 nm or 1 µm diameter) was formed on a silicon wafer as a lithographic mask. Through this mask, chromium was evaporated to form a 1–2 nm thick adhesive layer, followed by gold, until a thickness of about 50 nm was obtained (Figure 2b). The colloids were then removed, so that a hexagonal pattern of gold triangles on silicon was obtained [30]. The lateral dimensions of the surface pattern could be easily adjusted by varying the colloid size. The gold islands were then reacted with **LS-BP**, and either **PSB** or **SMAMP** were spin coated onto the surface and UV-irradiated at 254 nm. Thus, polymer-functionalized gold islands on a silicon background were obtained (named **PSB@Au_Si** or **SMAMP@Au_Si**, respectively, see Figure 2b). In order to functionalize the remaining silicon patches, they were reacted with **3EBP**. Next, the same polymer as in the previous step was spin-coated onto the surface, and UV irradiated. This gave samples with a polymer monolayer on top of a periodically structured substrate (**SMAMP@Au_SMAMP@Si** or **PSB@Au_PSB@Si**, respectively, Figure 2b). (The chemical contrast of Au on Si, for which this process was originally designed [30] was not strictly necessary to fabricate these materials, i.e., we could have also evaporated gold islands onto a gold substrate, or SiO_2_ onto silicon. The polymer monolayer would then have formed in one step. The two step method was used for two reasons: first, it gave access to the partially functionalized intermediates (**PSB@Au_Si** and **SMAMP@Au_Si**, respectively), and second, keeping the process the same made the results consistent with and comparable to previous data [30]).

### 2.2. Physical Surface Characterization

#### 2.2.1. Atomic Force Microscopy

**Figure 3:**
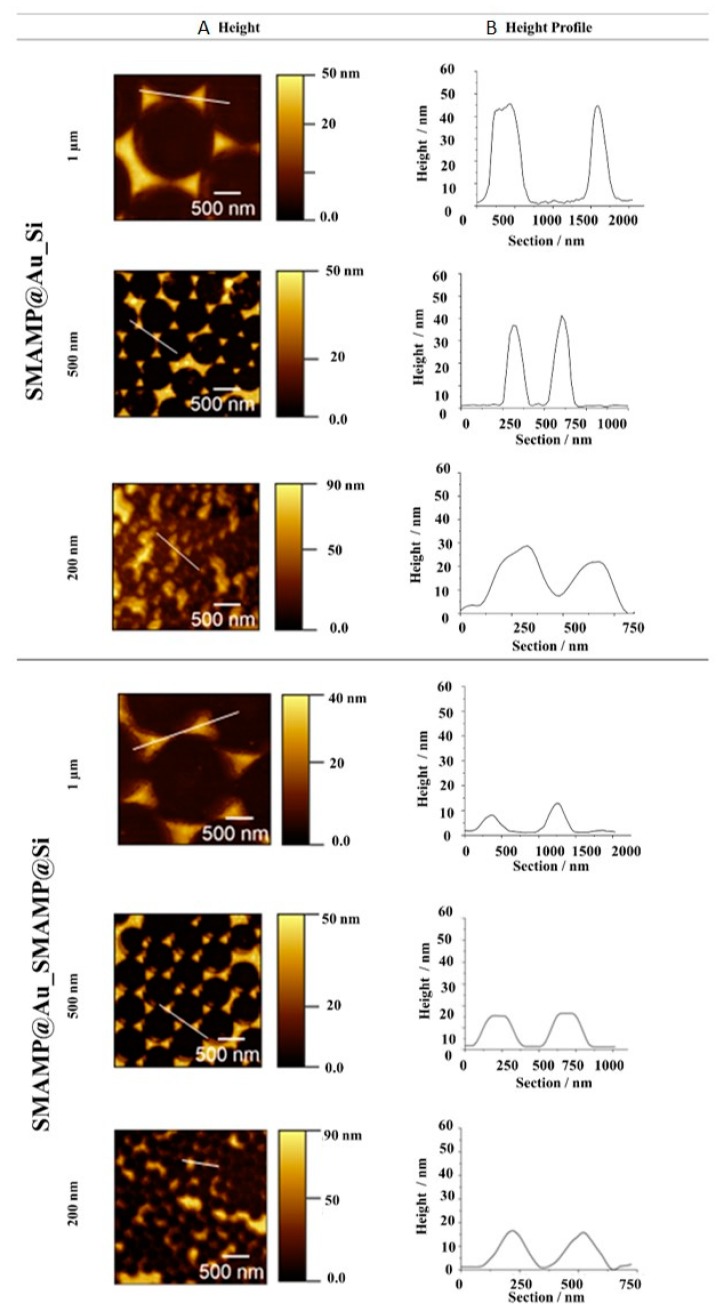
Atomic force microscopy (AFM) height images (**A**) and height profiles (**B**) of **SMAMP@Au_Si** and **SMAMP@Au_SMAMP@Si** structures with 1 µm, 500 nm and 200 nm spacing. The shifted hexagonal pattern in two samples is caused by a small misalignment of the Cr and Au sources during metal deposition. However, the samples were stable despite this mismatch, and the characteristic dimensions of the surfaces were not substantially altered.

**Figure 4:**
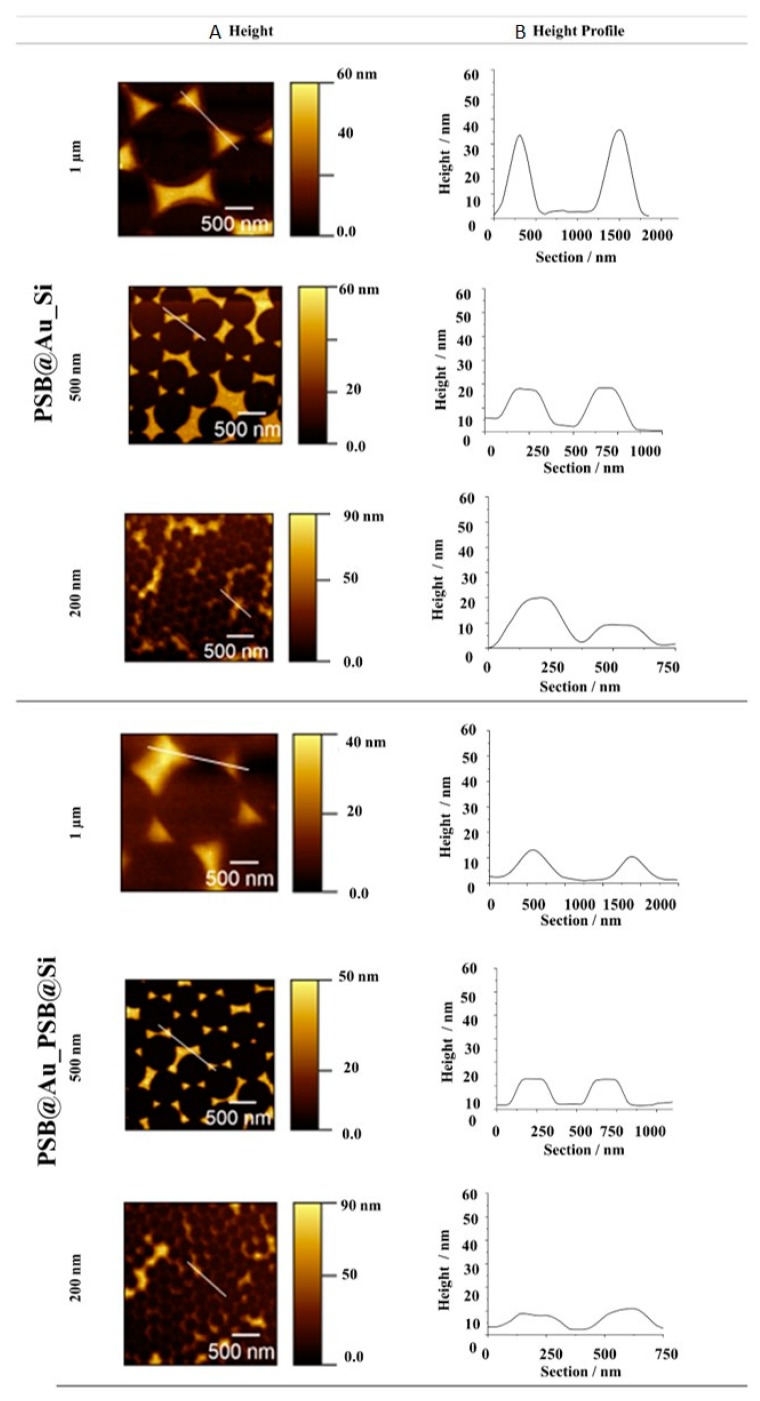
AFM height images (**A**) and height profiles (**B**) of **PSB@Au_Si** and **PSB@Au_PSB@Si** structures with 1 µm, 500 nm and 200 nm spacing.

Atomic Force Microscopy (AFM) height images were recorded after each polymer immobilization step (Figure 3 and Figure 4). The images document that the polymer- functionalized gold islands of **PSB@Au_Si** and **SMAMP@Au_Si** had slightly blurred domain edges, as gold-bound polymer chains spilled over onto the silicon background. The height of these mono-functionalized islands was about 30 to 45 nm depending on the sample spacing (200 nm to 1 µm). This is consistent with previous results [30]. For the “backfilled” surfaces with twofold polymer functionalization (**PSB@Au_PSB@Si** and **SMAMP@Au_SMAMP@Si**), the domain edges became smoother. The height profiles show a decrease in distance (z-direction) between the island peaks and the background to about 15 to 35 nm. This confirms that polymer was immobilized on Si background. Still, the underlying surface pattern was visible, as the attached polymer only had the thickness of about one monolayer.

#### 2.2.2. Contact Angle Measurements

To further study the effects of structuring and chemical functionalization, the static, advancing and receding and contact angles were measured, and compared to those of unstructured reference surfaces (blank **Si** wafer, **PSB** and **SMAMP monolayer**, Table 1).

The **Au_Si** samples with the smaller spacing had higher advancing contact angles than **Si**, as predicted by the Cassie-Baxter model, but these came down to similar values as the blank **Si** with increasing spacing. For the partially polymer-functionalized samples **PSB@Au_Si** and **SMAMP@Au_Si**, the contact angles decreased compared to the **Au_Si** surfaces, but not to values as low as the parent PSB and SMAMP monolayers. This is to be expected in the light of the bare silicon patches that were still present on these samples. The fully polymer-functionalized materials had overall similar contact angles as their parent monolayers, with a trend towards smaller values for smaller spacings.

#### 2.2.3. Protein Adhesion Studies by Surface Plasmon Resonance Spectroscopy

For surface plasmon resonance spectroscopy (SPR), gold substrates are needed so that surface plasmons can be excited [18]. Thus, lanthan fluoride glass slides onto which a gold layer had been evaporated were used as substrate for the SPR samples. To obtain similarly structured materials on gold, the fabrication process was ‘reversed’, insofar as silicon oxide was evaporated through the colloidal masks onto a gold background, yielding **SiO_2__Au** samples. These were treated with the **3EBP** linker and functionalized with the first polymer to obtain **PSB@SiO_2__Au** and **SMAMP@SiO_2__Au**, respectively. The gold patches were reacted with **LS-BP** and then polymer-functionalized [30], so that **PSB@SiO_2__PSB@Au** and **SMAMP@SiO_2__SMAMP@Au** were obtained.

**Figure 5:**
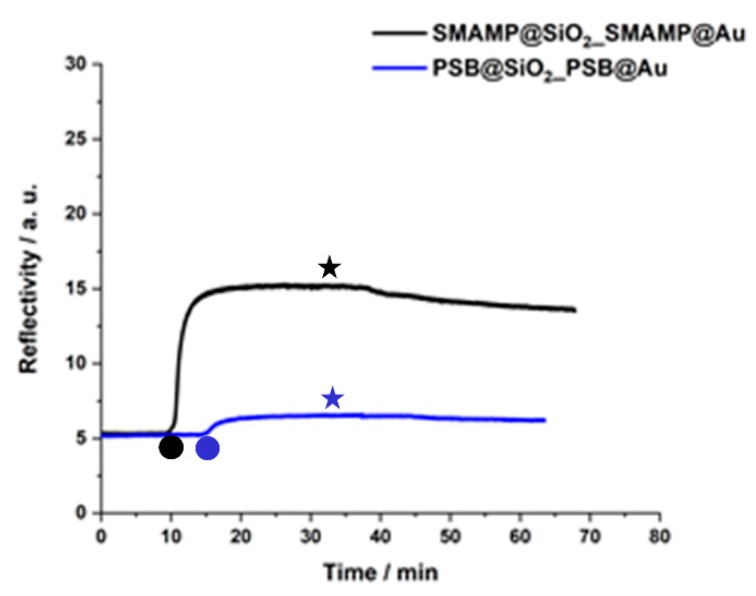
SPR kinetics curves of fibrinogen adhesion on **PSB@SiO_2__PSB@Au** and **SMAMP@SiO_2__SMAMP@Au**, both with 500 nm spacing. Circles: time point of protein injection; stars: time points of buffer injection.

As protein adhesion is the first step when surfaces interact with biological fluids, we used SPR to study the interaction of the structured and functionalized surfaces with fibrinogen. The samples were exposed to the protein in a flow cell, and full angular SPR scans of the dry samples before and after protein adhesion were taken. Additionally, the kinetics of protein adhesion was monitored in situ (Figure 5). Both procedures have been described in detail previously [17,30,32]. As these experiments are quite involved and sample preparation is costly, SPR data was obtained only for the samples with the 500 nm spacing. Figure 5 shows the kinetics of fibrinogen adhesion on the structured **PSB@SiO_2__PSB@Au** and **SMAMP@SiO_2__SMAMP@Au** samples. The data qualitatively shows that a significant amount of protein adhered to the SMAMP-functionalized surface, while the protein adhesion on the PSB-functionalized surface was much lower.

The dry full scans before and after protein exposure were fitted with the Fresnel equations to obtain their layer thickness and refractive index. From the thickness difference before and after protein adhesion, the amount of adhered protein was quantified as mass per area (m A^−1^), using ρ = m V^−1^ and V= t A, where ρ = protein density, m = protein mass, V = protein volume, t = thickness, A = surface area and ρ_fibrinogen_ = 1.085 g cm^−3^ [35]. The results for the structured **PSB@SiO_2__PSB@Au** and **SMAMP@SiO_2__SMAMP@Au** surfaces were compared to the plain PSB and SMAMP monolayers, the non-functionalized **SiO_2__Au** samples, and the partially polymer-functionalized **PSB@SiO_2__Au** and **SMAMP@SiO_2__Au** samples (Table 2). The SPR data obtained revealed that the structured **PSB@SiO_2__PSB@Au** surfaces were strongly protein-repellent. Since previous SPR data from PSB monolayers showed that the monolayers were not protein-repellent, probably due to surface dewetting during the layer fabrication, this indicates that dewetting was not a problem in the fabrication of the structured materials. This is in line with the AFM data for the structured surfaces (Figure 3 and Figure 4), which showed that the functionalized domains were covered with the respective polymer. Thus, the intrinsic protein-repellency of the polyzwitterionic PSB, which was previously observed for PSB networks with full surface coverage [17,36], was also retained in the structured materials here presented. Interestingly, the partially functionalized **PSB@SiO_2__Au** sample was already strongly protein-repellent (0.2 ng mm^−2^). Apparently, the spill-over of PSB from the SiO_2_ islands also affected adhesion to the Au background. Coverage of the surfaces with adhesive SMAMP, on the other hand, markedly affected protein adhesion: it was higher on the fully SMAMP-covered **SMAMP@SiO_2__SMAMP@Au** (8.0 ng mm^−2^) than on the partially SMAMP-covered **SMAMP@SiO_2__Au** (2.8 ng mm^−2^), which is comparable to protein adhesion on the SMAMP-less **SiO_2__Au**. Nevertheless, the protein adhesion on **SMAMP@SiO_2__SMAMP@Au** was lower than on the **SMAMP** monolayer (13 ng mm^−2^). Apparently, the underlying microstructure helped reduce protein adhesion. This assumption is in line with the data from the **SiO_2__Au** surface, which was also somewhat protein-repellent (3.7 ng mm^−2^).

### 2.3. Biological Surface Characterization

**Figure 6:**
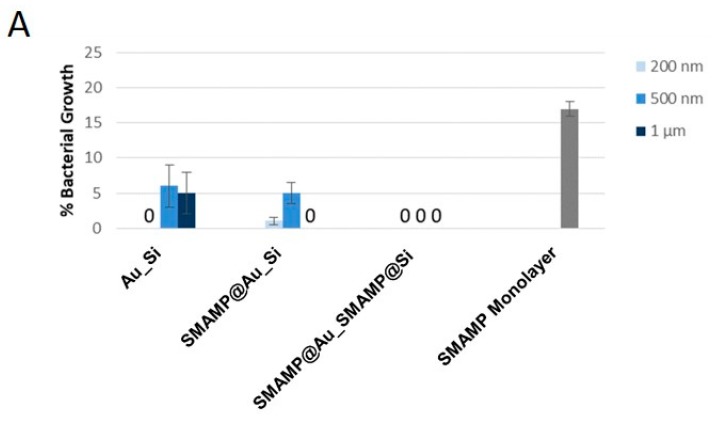

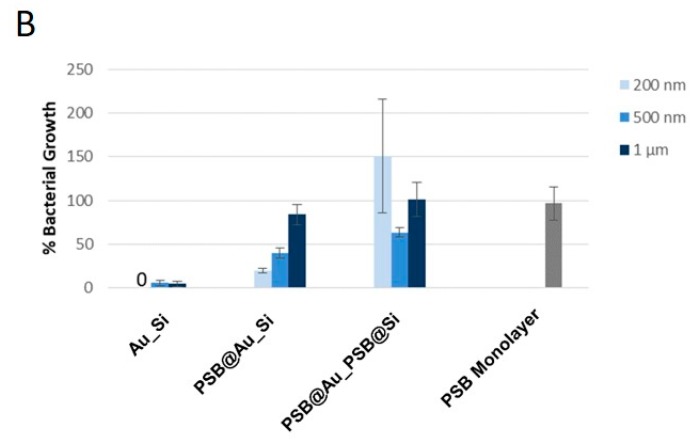
Antimicrobial activity of the structured and functionalized surfaces against *E. coli* bacteria. The normalized percentage of surviving bacteria was plotted each sample type. (**A**) SMAMP-functionalized samples, (**B**) PSB-functionalized surfaces, together with reference materials. **Au_Si**, **SMAMP@Au_Si**, **PSB@Au_Si** and the monolayer controls from [30]. The error bars indicate the standard deviation calculated from two independent experiments with 5 replicas each.

The antimicrobial properties of the above presented surfaces against *Escherichia coli* bacteria were quantified with a standardized antimicrobial assay as described in the Experimental Section [33]. Briefly, the surfaces were sprayed with a suspension of *E. coli* (10^6^ bacteria mL^−1^) and incubated for 4 °h, after which and the surviving bacteria (colony forming units, CFUs) were counted. Reduction of CFUs in this assay points to an antimicrobial action of the substrate, i.e., the reduction of the number of bacteria present on the surface compared to an inactive control surface. The percentage of CFUs on the test surfaces normalized to the growth control with spacings from 200 nm to 1 µm is plotted in Figure 6. As reported previously [30], the surface structure itself (**Au_Si**) strongly inhibited bacterial growth at all spacings even in the absence of an intrinsic antimicrobial activity of either gold or silicon. This is most likely a consequence of reduced bacterial adhesion on the structures, as was observed previously on other structured systems [4,37]. The **SMAMP@Si_SMAMP@Au** surfaces on the other hand quantitatively killed *E. coli* bacteria at all spacings due to the intrinsic antimicrobial effect of the **SMAMP** polymer. These materials even outperformed the **SMAMP** monolayer, which had 17% CFUs. For the partially functionalized **SMAMP@Au_Si** surfaces, substantial antimicrobial activity was observed, with quantitative killing at a spacing of 1 µm. As expected, the antimicrobial activity of the **PSB**-functionalized surfaces was low across all spacings for **PSB@Au_Si** and even lower for the **PSB@Au_PSB@Si** samples. Interestingly, a trend of increasing bacterial growth with increasing spacing was observed for the **PSB@Au_Si** series. For the **PSB@Au_PSB@Si** series, a similar trend is seen (the data point at 200 nm has an unusually large error bar, potentially due to a large number of surface defects, and will therefore be disregarded). Both observations probably are the effect of the underlying surface pattern. The smaller the spacing, the more effectively bacterial adhesion is prevented. Apparently, bacterial adhesion is not as effectively reduced by **PSB** as by the bare **Au_Si** surfaces, and since neither the polymer nor the substrate have intrinsic antimicrobial activity, the CFU count increases with increasing size of the **PSB** patches.

**Figure 7:**
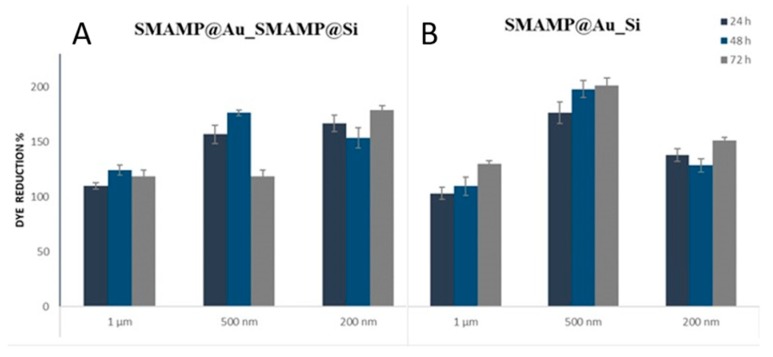

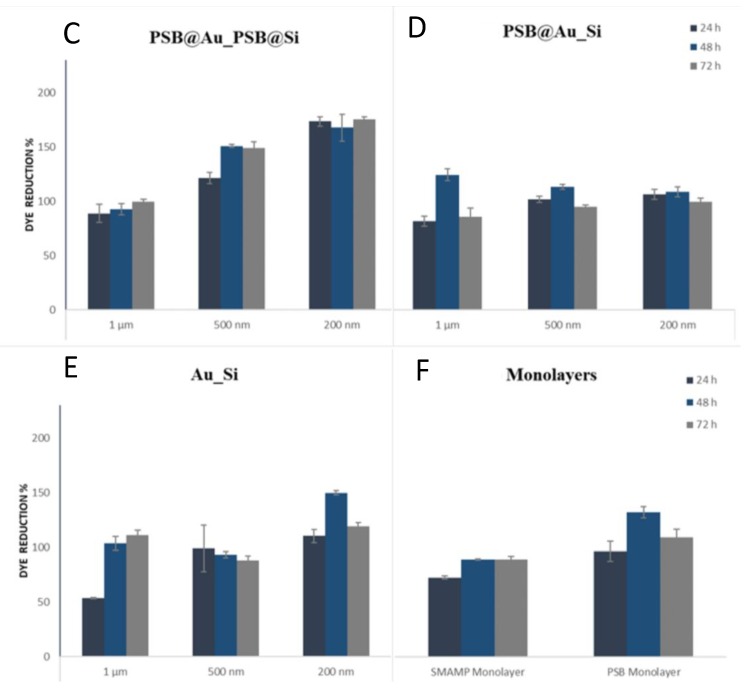
Normalized relative dye reduction (in%) of keratinocytes grown on the structured and functionalized samples: (**A**) **SMAMP@Au_SMAMP@Si**, (**B**) **SMAMP@Au_Si**, (**C**) **PSB@Au_PSB@Si**, (**D**) **PSB@Au_Si**, (**E**) **Au_Si**, and (**F**) monolayers. The error bars indicate the standard deviation calculated from two independent experiments with 3 replicas each.

The compatibility of the structured surfaces with human gingival mucosal keratinocyte (GM-K) cells was evaluated using three methods. First, the morphology and density of keratinocytes grown on the surfaces was examined with optical microscopy (phase contrast). The ratio of healthy and membrane-compromised keratinocytes was qualitatively visualized with a live-dead assay for mammalian cells (using the green-fluorescent SYTO16 and the red-fluorescent propidium iodide stains). The Alamar Blue assay was used to quantify the metabolic activity of immortalized human keratinocytes cultured on the surfaces. In this assay, the reduction of the resazurin dye is monitored as an indicator for cell activity. The Alamar Blue assay was performed by seeding immortalized, non-carcinogenic GM-K cells onto the test surfaces and cultivating them for 24, 48 and 72 h, respectively. The dye reduction at these time points was measured and normalized to the dye reduction of the growth control (blank, uncoated glass substrates). The data (normalized to the growth control) is shown in Figure 7.

The Alamar Blue data shows that none of the tested surfaces was toxic to the GM-K cells, as none of the materials caused an excessive reduction in cell metabolism. Likewise, the maximal dye reduction was at ~200%, which is a significant increase but not a sign of out-of-control proliferation. A significantly lower growth rate (relative to the respective growth controls) was observed for the **PSB@Au_Si** series. On these materials, the dye reduction was about as low as on the **Au_Si** surfaces, which is an indication that the underlying pattern, which was only partially polymer-covered, also reduces cellular growth on these materials. The cell metabolism on the **PSB@Au_Si** and the **PSB@Au_PSB@Si** samples at 1 µm spacing was equally low, yet in the dye reduction increased to > 160% for the two **PSB@Au_PSB@Si** samples with smaller spacings. A similar trend of increased dye reduction with smaller sample spacing is seen in the **SMAMP@Au_SMAMP@Si** data. Overall, the dye reduction is significantly higher for the **SMAMP** samples than for the corresponding **PSB** samples. The samples of the **SMAMP@Au_Si** series are at a similarly high level of dye reduction as the **SMAMP@Au_SMAMP@Si** samples, yet the maximum was observed for the 500 nm spacing and not the 200 nm spacing. This sample also had the highest dye reduction of all materials tested.

**Figure 8:**
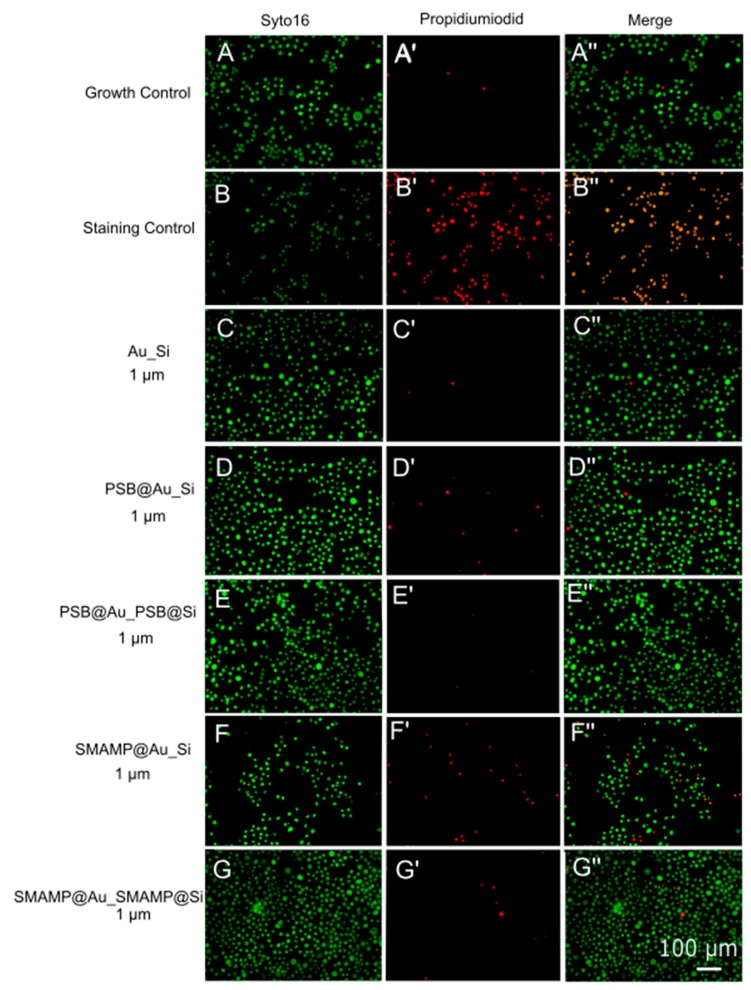
Live dead stain images of keratinocytes grown on structured and polymer-functionalized surfaces. Syto16 (green, left colum) stained “living” cells and propidium iodide (red, middle column) stained “dead” (= membrane-compromised) cells. (**A**, **A’**, **A’’**) growth control, cells grown on glass); (**B**, **B’**, **B’’**) staining control (cells grown on glass treated with isopropanol); (**C**, **C’**, **C’’**) **Au_Si**; (**D**, **D’**, **D’’**) **PSB@Au_Si**; (**E**, **E’**, **E’’**) **PSB@Au_PSB@Si**; (**F**, **F’**, **F’’**) **SMAMP@Au_Si**, and (**G**, **G’**, **G’’**) **SMAMP@Au_SMAMP@Si**. Images with plain letters (**A**–**G**, left column) were taken using a GFP filter for green fluorescence; images with one dash (**A’** to **G’**, middle column) were taken using a TexasRed filter for red fluorescence. The images with two dashes (**A’’** to **G’’**, right column) are a digital overlay of the red- and green-fluorescent images.

Optical microscopy images (Figure S1 of the Supporting Information) showed no significant differences between the morphology of keratinocytes grown on **PSB@Au_Si**, **PSB@Au_PSB@Si**, **SMAMP@Au_Si** or **SMAMP@Au_SMAMP@Si** surfaces, and the growth control. This was further confirmed with fluorescence microscopy, showing that most of the cells grown on the functionalized surfaces were viable (green) and only few keratinocytes were found membrane-compromised (red, shown in Figure 8 for the samples with 1 µm spacing). These Live-Dead-images for all samples can be found in Figure S2 in the Supporting Information.

## 3. Discussion

The above-described data is strong evidence that surface structuring combined with chemical surface functionalization is an effective tool to manipulate the interaction of surfaces with biological entities. So far, only few reports exist on simultaneously structured and chemically modified bioactive surfaces [30,38]. Thus, it is difficult to differentiate whether protein adhesion, antimicrobial activity and cell compatibility are more strongly influence by any surface structuring, or by chemical functionalization. In a previous publication [30], we investigated samples with mixed functionalities (i.e., **SMAMP@Au_PSB@SiO_2_** and **PSB@Au_SMAMP@SiO_2_**). By comparing these results with the here presented data, a quite detailed picture can now be painted for the here presented system. First, protein adhesion was near-quantitatively reduced at any spacing as long as **PSB** was present on the surface. For the sample series with 500 nm spacing, both **SMAMP@SiO_2__PSB@Au** and **PSB@SiO_2__SMAMP@Au** showed near-quantitative reduction of protein adhesion [30], and so did the here presented **PSB@SiO_2__PSB@Au** and **PSB@SiO_2__Au** samples. We have previously shown that surface structuring itself reduced protein adhesion [30], but not as quantitatively as additional **PSB**. On the other hand, the cationic **SMAMP@Au_SMAMP@SiO_2_** surfaces were strongly protein-adhesive, yet less so than a **SMAMP** monolayer. This can also be related to the presence of the underlying surface structure. Thus, an underlying surface structure in the range of 200 nm to 1 µm reduced protein adhesion in the here presented sample set. The finding that the **SMAMP@Au_SMAMP@Si** materials were fully antimicrobial, even more strongly so than a **SMAMP** monolayer, could also be related to the underlying structure. Bacteria are rather rigid and cannot deform easily, which might have limited the contact area of bacteria with the SMAMP monolayer. The underlying structure might have increased that contact area in the case of the **SMAMP@Au_SMAMP@Si** samples, making them overall more bactericidal than the monolayer. Interestingly, both the **Au_Si** and the **PSB@Au_PSB@Si** samples - with no intrinisic antimicrobial activity of either substrate or polymer - showed CFU reduction due to the underlying surface structure, which prevented bacterial adhesion. Yet at larger spacings, when the bacteria were the same size as the structures, bacterial adhesion could no longer be inhibited. Many reports have shown that structured surfaces with feature dimensions that are equally sized or smaller than bacteria successfully reduce bacterial adhesion [39]. Our findings support this theory.

Additionally, we observed with the Alamar Blue assay data that an underlying surface structure increased the cell growth with decreasing spacing in the here presented system, and that this effect was further enhanced either by the presence of adhesive **SMAMP** patches, or by full surface coverage with **PSB**. Thus, we demonstrated that combined surface structuring and chemical functionalization can significantly enhance cellular growth and proliferation, presumably through improved cell adhesion. This is particularly relevant in the context of tissue integration.

The interaction of structured surfaces with cells are not yet fully understood, yet it is known that cells attach to a surface through integrins and form so-called focal points by clustering of integrin receptors on the cell surface, followed by recruiting of integrins from the cytoplasm. Integrins are small (<12 nm), while the focal points are rather large (typically 1–5 µm)—thus it is considered plausible that surface structures with length scales both in the nano- and the micrometer range will affect cell adhesion [21]. They could either interfere with the integrin adhesion to the surface, the focal point formation, cellular motion, or affect the cellular shape; by signal transduction mechanisms including mechano-transduction, these effects might ultimately even influence gene expression [21]. Thus, the trend observed that smaller spacings enhance cellular adhesion could be related to a change in the protein composition on theses surfaces in favor of the integrins. However, this hypothesis needs to be verified by further experiments. The here presented materials contain patches of cationic polymers, which are known to enhance cell adhesion to surfaces [40]. Interestingly though, cationic polymer alone does not improve the cell adhesion compared to a growth control surface, as seen on the **SMAMP** monolayer. The synergy with the surface structure, however, leads to the highest cell growth rates observed in the sample set. This data is an indication that the two synergistic effects—surface patterning and adhesive polymer patches—might lead to improved tissue integration.

## 4. Materials and Methods

### 4.1. Surface Structuring and Functionalization

The polymer synthesis, surface structuring process and surface functionalization have been described previously [30]. Briefly, a colloidal monolayer made from 200 nm, 500 nm and 1 μm polystyrene beads (obtained from Micromod GmbH, Rostock, Germany) was transferred to a silicon wafer piece and served as a lithographic mask in the subsequent evaporation of 5 nm chromium and 40 nm gold. After colloidal mask lift-off, the resulting contrasting gold-silicon surface was site-selectively functionalized with UV-reactive linker molecules: a 20 mg mL^−1^ solution of **3EBP** in toluene was spin-coated (at 3000 rpm for 30 s) onto the substrate and cured at 120 °C for 30 min to selectively functionalize the silane patches. After rinsing with toluene, the desired polymer was spin coated onto the surface. For this, 10 mg mL^−1^ solutions of 100,000 g mol^−1^ **SMAMP** in dichloromethane or 50,000 g mol^−1^ **PSB** in trifluoroethanol were used. The substrates were UV-irradiated (total irradiation energy: 3 J) to covalently attach the polymer to the surface, and unbound polymer was removed by washing. The gold islands were functionalized with (**LS-BP**, 5 mmol mL^−1^ LS-BP in toluene) by incubation for 15 h. The gold islands were polymer-covered analogously to the silane patches using the same polymer solutions. Finally, the **SMAMP** functionalized surfaces were deprotected by immersion in 4 M HCl in dioxane overnight.

### 4.2. Surface Characterization

All physical characterization techniques were performed as reported previously [30]. Brief details are given below.

#### 4.2.1. Atomic Force Microscopy

Topographical images of the surfaces were recorded with a Dimension Icon from Bruker (Karlsruhe, Germany). The measurements were performed in ScanAsyst mode and commercial ScanAsyst Air cantilevers were used for all measurements. The attained imaged were processed with the software ‘Nanoscope Analysis 1.5′ from Bruker.

#### 4.2.2. Contact Angle Measurements

The static, advancing and receding contact angles were measured at five different locations on each sample and the average was reported.

#### 4.2.3. Surface Plasmon Resonance Spectroscopy

To study the protein adhesion on the surfaces, surface plasmon spectroscopy was performed in kinetics mode on a RT2005 RES-TEC device (Res-Tec, Framersheim, Germany) in Kretschmann configuration. Full reflectivity scans were recorded before and after each experiment and the thickness of each sample was calculated based on the Fresnel equations with the software ‘Winspall’ (Version 3.02, Res-Tec, Framersheim, Germany).

### 4.3. Biological Assays

#### 4.3.1. Antimicrobial Activity Assay

The antimicrobial activity of the surfaces was analyzed with a modification of the Japanese Industrial Standard JIS Z 2801:2000 ‘Antibacterial Products Test for Antibacterial Activity and Efficacy’, as reported previously [30]. Briefly, *E. coli* (ATCC25922) was cultured overnight in tryptic soy broth and diluted by 1:10. After 3–4 h, the optical density was checked and 150 µL of the *E. coli* bacterial culture was mixed with 100 mL of 0.9% sterile NaCl solution in a chromatography spray bottle under continuous stirring. All test samples (including positive and negative controls) were placed centrally on a sterile Petri dish, which was positioned at a distance of 15 cm to the spray nozzle. Then, the bacterial suspension was sprayed on the samples using compressed air from a 50 mL syringe. The petri dishes were covered and incubated for 2 h in a humid chamber. Next, 50 µL of sterile 0.9% NaCl solution was placed on the samples for 2 min, then the bacterial dispersion was aspirated with a pipette and spread over Columbia blood agar plates. These surfaces were incubated over night at 37 °C without agitation. The number of colony forming units (CFUs) was counted with the software ‘Quantity One’ and each experiment was performed at least twice. The growth percentage was calculated via Equation (1):

% growth = (CFUs_sample_ − CFUs_positive control_)/(CFUs_negative control_ − CFUs_positive control_) × 100 (1)

#### 4.3.2. Optical Microscopy and Alamar Blue Assay

*Ethics Statement*: Gingival mucosal keratinocytes were obtained from human volunteers who previously signed their consent according to the Helsinki declaration. This was approved by the Ethics Board of the Albert-Ludwigs University Freiburg, Germany (ethics vote number 381/15).

Alamar Blue assays were performed in triplicates as reported previously [17]. In short, glass coverslips with 22 mm diameter and a thickness of 0.5 mm (Langenbrick, Emmendingen, Germany) were structured and functionalized as described for the silicon substrates. Blank coverslips were used as controls and washed for 30 min in 100% isopropanol. All test samples were sterilized for 15 min in 70% ethanol; residual ethanol was removed by washing all coverslips (both test and control samples) three times with PBS buffer. For the assay, the coverslips were placed in 12-well plates (bio-one Cellstar, Greiner, Frickenhausen, Germany). Gingival mucosal keratinocytes (GM-K) (immortalized using HPV-16) were cultured in keratinocyte growth medium (KGM, Promocell, Heidelberg, Germany) with accompanying supplement concentrations as supplied by the manufacturer: bovine pituitary extract: 0.004 mg mL^−1^; epidermal growth factor (EGF): 0.125 ng mL^−1^; insulin: 5 μg mL^−1^; hydrocortisone: 0.33 μg mL^−1^; epinephrine: 0.39 μg mL^−1^; transferrin: 10 μg mL^−1^; CaCl_2_, 0.06 mM and the antibiotic kanamycin at 100 μg mL^−1^. At a confluency of 70–90%, the cells were detached with accutase (Sigma-Aldrich, Munich, Germany) and resuspended in supplement/antibiotic-free KGM. The cells (1 mL of cell dispersion with a concentration of 1.5 × 10^5^ cells mL^−1^ in supplement/ antibiotic free medium) were then seeded out onto the samples. The well plates were incubated at 37 °C / 5% CO_2_ for 5 h to enable settlement and adhesion to the coverslips. Afterwards, 500 µL of the supernatant medium was carefully aspirated and replaced by 500 µL of fresh medium with the double supplement concentration, yielding the initial supplement medium concentration. For time dependent analysis, the cells were cultivated for 18 more hours (total 24 h), 42 h (total 48 h) and 66 h (total 72 h), respectively. Positive and negative controls were generated at each time point: For positive controls, 500 µL of medium were aspirated from three wells and 500 µL of fresh medium and 60% isopropanol were added, yielding a 30% isopropanol solution. For the negative controls, 1 mL medium was removed and replaced by 1 mL of fresh medium. Optical micrographs of the samples with grown keratinocytes were recorded at 200x magnification with a Leica DMIL microscope with a Leica D-LUX-3 CCD camera. For the Alamar blue assay, all samples and controls were cultivated for additional 30 min. Afterwards, 110 µL of Alamar Blue (AbD Serotec, Oxford, UK, pre-warmed to 37 °C) was slowly pipetted into the wells, yielding a 10% solution. The wells were gently agitated to provide a homogeneous dispersion. After re-incubating the cells for 2 h, the supernatant of each well was aspirated and collected in a 1.5 mL Eppendorf tube. The tubes were centrifuged at 1000 g for 5 min. Then, the fluorescence intensity of the supernatant was measured on a Tecan Infinte 200 plate reader (excitation at 540 nm; measurement at 590 nm). The collected data was analyzed according to the Alamar Blue manufacturer’s instructions.

#### 4.3.3. Live-Dead Staining of Keratinocytes Grown on Structured Functionalized Surfaces

Live-dead staining assays were performed after cultivating the keratinocytes for 72 h in the Alamar Blue assay. Instantly after the removal of supernatant, the GM-Ks were washed twice with PBS. Syto16 green fluorescent nucleic acid stain (Molecular Probes, Eugene, OR, USA), diluted 1:200 in keratinocyte growth medium (Promo Cell, Heidelberg, Germany) was used for live staining, while propidium iodide (Sigma-Aldrich GmbH, Steinheim, Germany; dilution 1:1000) was used for the dead cell staining. The GM-K cells were incubated with the stains for 30 min at 37 °C in humidified air containing 5% CO_2_ and then washed with PBS twice. The cell viability was determined in PBS directly afterwards. Images of the samples were analyzed and recorded with a Keyence BZ-9000E florescence microscope with the software BZ II Viewer and BZ II Analyser. Green fluorescence was measured at ca. 490 nm excitation and red at 536 nm, respectively. The image contrast was adjusted for a better visualization.

## 5. Conclusions

In this work, we tested the combined effects of surface topography and chemical surface functionalization on the interaction of a material with proteins, bacteria and mammalian cells. To do so, an underlying surface pattern with lateral dimensions between 200 nm and 1 µm and a height between 15–50 nm was fabricated using colloidal lithography, and either partially or fully functionalized with antimicrobial, cell-adhesive polycations or protein-repellent polyzwitterions. These materials were compared to the non-functionalized structured surfaces and unstructured polymer monolayers. While the data showed that the underlying structure reduced protein adhesion and bacterial adhesion, it enhanced cellular adhesion, particularly at small spacings. This effect was particularly pronounced when the surface pattern was combined with cell-adhesive polycations. Thus, materials with very high antimicrobial activity, reduced unspecific protein adhesion, and enhanced cellular adhesion were obtained. Functionalizing structured surfaces with adhesive polymer could thus be a valuable tool for improved tissue integration of implants. However, before this claim can be made, further systems with similar surface features should be studied, and the performance of the materials needs to be studied in animal models.

## Figures and Tables

**Table 1:** Contact angle data (static, advancing and receding contact angles) for the homogeneous and structured polymer surfaces. Contact angles of **Si**, the polymer monolayers, **PSB@Au_Si** and **SMAMP@Au_Si** have been previously published [30].

Sample Type	Contact Angle (°)		
	*θ_static_*	*θ_advancing_*	*θ_receding_*
**Si**	71 ± 1	75 ± 3	41 ± 3
**PSB monolayer**	34 ± 3	35 ± 3	22 ± 1
**SMAMP monolayer**	59 ± 3	61 ± 3	33 ± 3
**Au_Si**			
200 nm	88 ± 3	91 ± 3	65 ± 3
500 nm	81 ± 1	86 ± 2	63 ± 1
1 µm	73 ± 4	74 ± 4	66 ± 2
**PSB@Au_Si**			
200 nm	38 ± 3	43 ± 2	31 ± 2
500 nm	53 ± 3	70 ± 1	42 ± 2
1 µm	51 ± 3	62 ± 2	43 ± 1
**PSB@Au_PSB@Si**			
200 nm	24 ± 3	28 ± 2	20 ± 1
500 nm	40 ± 3	36 ± 3	27 ± 2
1 µm	31 ± 4	33 ± 3	25 ± 4
**SMAMP@Au_Si**			
200 nm	56 ± 3	55 ± 3	33 ± 2
500 nm	60 ± 2	69 ± 2	45 ± 1
1 µm	63 ± 3	71 ± 4	47 ± 3
**SMAMP@Au_SMAMP@Si**			
200 nm	48 ± 1	42 ± 4	29 ± 4
500 nm	43 ± 3	59 ± 3	35 ± 3
1 µm	65 ± 1	68 ± 4	45 ± 4

**Table 2:** Average amount of fibrinogen (in ng mm^−2^) adhered on the structured materials. Part of the data was previously reported [30].

Sample	Adhered Fibrinogen (ng mm^−2^)
**PSB monolayer**	11
**SiO_2__Au**, 500 nm	3.7
**PSB@SiO_2__Au**, 500 nm	0.2
**PSB@SiO_2__PSB@Au**, 500 nm	0.0
**SMAMP monolayer**	13
**SMAMP@SiO_2__Au**, 500 nm	2.8
**SMAMP@SiO_2__SMAMP@Au**, 500 nm	8.0

## Supplementary Materials

The following are available online at https://www.mdpi.com/1420-3049/24/5/909/s1, Figure S1: Live-dead staining images of human keratinocytes (GM-K) grown on surfaces 200 nm to 1 µm spacing; Figure S2: Optical micrographs of human keratinocytes (GM-K) grown on surfaces 200 nm to 1 µm spacing.
