# Supplementary material for: Surface Structuring Combined with Chemical Surface Functionalization: An Effective Tool to Manipulate Cell Adhesion
Source: Molecules. 2019 Mar 5;24(5):909. doi: 10.3390/molecules24050909 (PMC6429452; doi:10.3390/molecules24050909)
Supplement: Supplementary file 1 [file molecules-24-00909-s001.pdf]

Article

Surface Structuring Combined with Chemical Surface Functionalization: An Effective Tool to Manipulate Cell Adhesion

Sarah M. Elsayed,[1,2] Stefan Paschke, [1,2] Sibylle Rau, [1,2] and Karen Lienkamp [1,2,*]

Freiburg Center for Interactive Materials and Bioinspired Technologies (FIT) and Department of Microsystems Engineering (IMTEK), Albert-Ludwigs-Universität, Georges-Köhler-Allee 105, 79110 Freiburg, Germany; sarah.mahmoud@imtek.uni-freiburg.de (S.M.E.), stefan.paschke@imtek.uni-freiburg.de (S.P.), sibylle.rau@uniklinik-freiburg.de (S.R.), lienkamp@imtek.uni-freiburg.de (K.L.)

*Correspondence: lienkamp@imtek.uni-freiburg.de; Tel.: +49-761-203-95090

Supporting Information

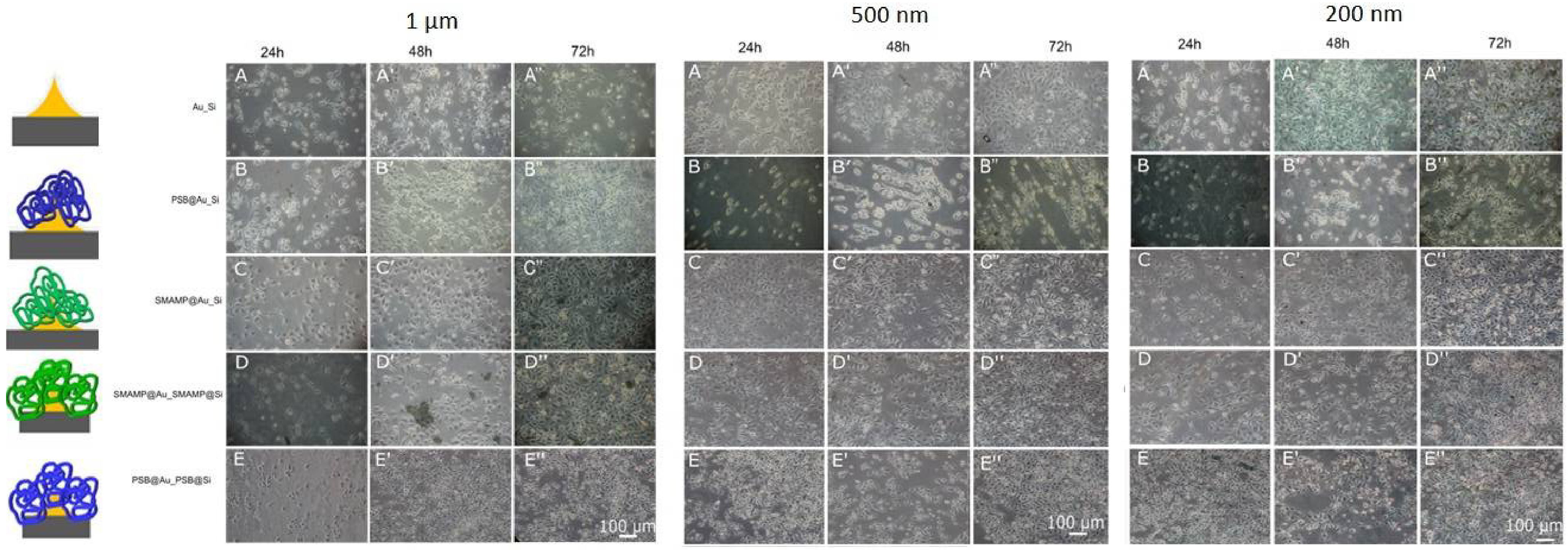

Figure S1: Optical micrographs of human keratinocytes (GM-K) after 24, 48 and 72 h growth on functionalized surfaces with 1 µm , 500 nm and 200 nm spacing.

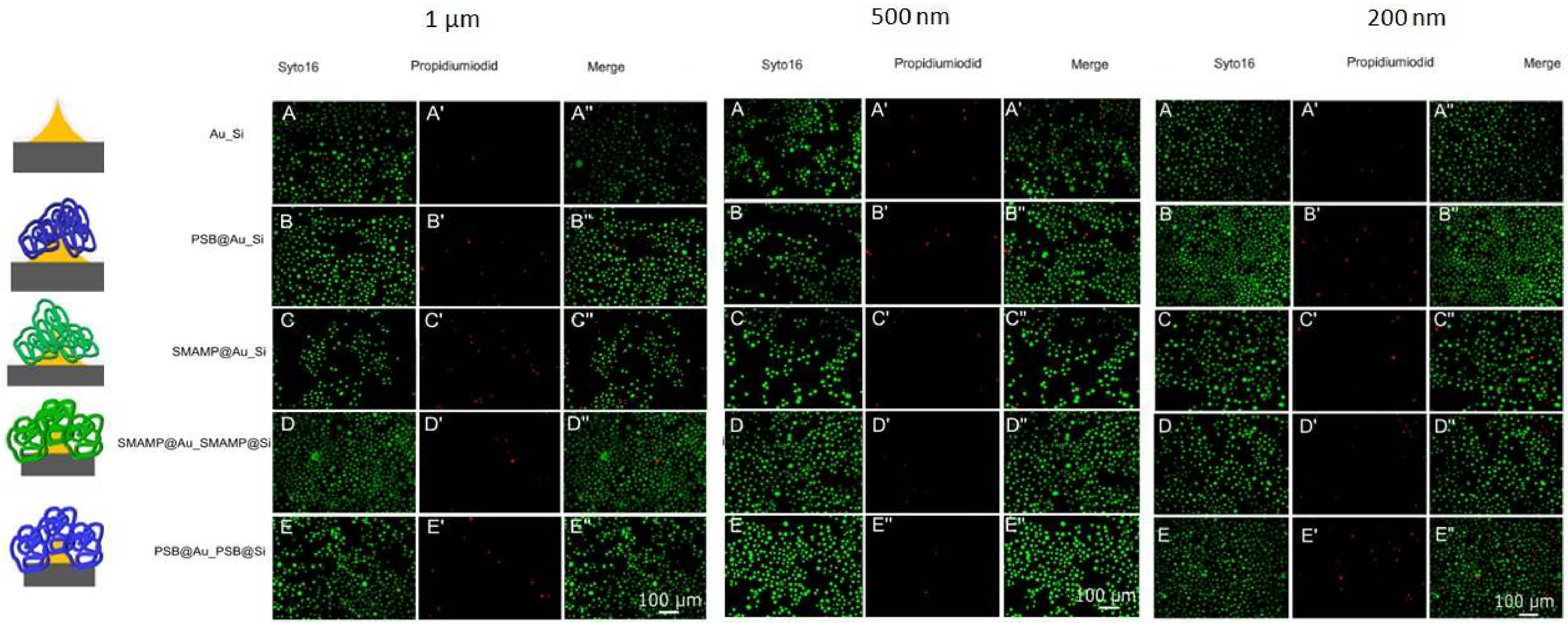

Figure S2: Live- dead staining images of human Keratinocytes (GM-K) after 72 h grown on functionalized surfaces with 1 µm , 500 nm and 200 nm spacing. The green stain (SYTO 16) visualizes live cells and the red stain (propidium iodide) the dead cells. Merged images are an overlay of both.
